# Circulating Levels of PD-L1 in Mesothelioma Patients from the NIBIT-MESO-1 Study: Correlation with Survival

**DOI:** 10.3390/cancers12020361

**Published:** 2020-02-05

**Authors:** Carla Chiarucci, Sara Cannito, Maria Grazia Daffinà, Giovanni Amato, Gianluca Giacobini, Ornella Cutaia, Maria Fortunata Lofiego, Carolina Fazio, Diana Giannarelli, Riccardo Danielli, Anna Maria Di Giacomo, Sandra Coral, Luana Calabrò, Michele Maio, Alessia Covre

**Affiliations:** 1 Center for Immuno-Oncology, Medical Oncology and Immunotherapy, Department of Oncology, University Hospital of Siena, 53100 Siena, Italy; carlabio@hotmail.it (C.C.); saracannito@hotmail.it (S.C.); mariagrazia.daffina@ao-siena.toscana.it (M.G.D.); dataman.immonco@gmail.com (G.A.); gianlucagiacobini@gmail.com (G.G.); cutaiaornella@gmail.com (O.C.); mariafl@hotmail.it (M.F.L.); carolinafazio137@gmail.com (C.F.); riccardo.danielli@ao-siena.toscana.it (R.D.); a.digiacomo@ao-siena.toscana.it (A.M.D.G.); scoral2@yahoo.it (S.C.); l.calabro@ao-siena.toscana.it (L.C.); mmaiocro@gmail.com (M.M.); 2 Unit of statistics, Regina Elena National Cancer Institute IRCCS, 00144 Rome, Italy; diana.giannarelli@gmail.com

**Keywords:** PD-L1, soluble PD-L1, cancer immunotherapy, immune checkpoint inhibitors, biomarker, mesothelioma

## Abstract

Targeting of the programmed cell death protein (PD)-1/programmed death-ligand 1 (PD-L1) axis has shown a significant clinical impact in several tumor types. Accordingly, our phase II NIBIT-MESO-1 study demonstrated an improved clinical efficacy in mesothelioma patients treated with the anti-PD-L1 durvalumab combined with the anti-cytotoxic T-lymphocyte antigen (CTLA)-4 tremelimumab, as compared to tremelimumab alone. Due to the promising therapeutic activity of immune check-point inhibitors (ICIs) in mesothelioma patients, the identification of biomarkers predictive of response to treatment is of crucial relevance. The prognostic role of soluble PD-L1 (sPD-L1) proposed in cancer patients prompted us to investigate this protein in sera from mesothelioma patients (*n* = 40) enrolled in the NIBIT-MESO-1 study. A significant (*p* < 0.001) increase in sPD-L1 levels was detected in patients after the first cycle and during therapy vs. baseline. A longer overall survival (OS) was observed in patients with sPD-L1 concentrations below (at baseline, d1C2, d1C5 (*p* < 0.01)) or FC values above (*p* < 0.05 at d1C2, d1C3, d1C5) their statistically calculated optimal cut-offs. On the basis of these initial results, the specific role of CTLA-4-, PD-L1-, or PD-1-targeting on sPD-L1 release was then investigated in sera from 81 additional ICI-treated solid cancer patients. Results showed a significant (*p* < 0.001) increase of sPD-L1 levels during therapy compared to baseline only in anti-PD-L1-treated patients, supporting the specific involvement of PD-L1 targeting in the release of its soluble form. Our findings suggest that sPD-L1 represents a predictive biomarker of clinical response to anti-PD-L1 cancer immunotherapy.

## 1. Introduction

The therapeutic relevance of monoclonal antibodies (mAbs) targeting inhibitory immune checkpoint(s) has been validated in a variety of tumor types [1]. Over the last years, several studies showed that patients with different malignancies, treated with mAbs blocking the programmed death (PD)-1/PD-1 ligand (PD-L1) pathway, obtained long term benefit with a good safety profile, compared to previously developed anti-cytotoxic T-lymphocyte antigen (CTLA)-4 mAbs [2,3]. PD-1/PD-L1 interaction prevents the suppression of effector T-cells proliferation and activation, inducing cytokine release and prompting the antitumor response [4,5,6]. Following the food and drug administration (FDA) approval of the anti-PD-1 mAbs nivolumab and pembrolizumab, improved knowledge of this pathway has led to the development and the subsequent FDA endorsement of additional immune checkpoint inhibitors (ICIs), such as the anti-PD-L1 mAbs atezolizumab, avelumab, and durvalumab [7]. An increasing number of clinical trials with different ICIs targeting PD-1/PD-L1 axis are ongoing in several tumor histotypes, alone or in combination with other immunotherapeutic or non-immunotherapeutic agents [8,9,10]. In this scenario, very limited clinical benefit was demonstrated in mesothelioma patients treated in monotherapy with the anti-CTLA-4 mAb tremelimumab or the PD-1/PD-L1 [11,12].

Promising results are emerging by the combined targeting of different immune checkpoint inhibitors. In particular the NIBIT-MESO-1 study was the first to demonstrate encouraging signs of efficacy from treatment with tremelimumab combined with durvalumab in first or second-line malignant mesothelioma patients [13]. Subsequently, similar results were also described in pre-treated pleural mesothelioma patients treated with ipilimumab plus nivolumab [14,15].

However, clinical efficacy of anti-PD-1/PD-L1 seems to depend on tumor intrinsic characteristics such as the neoantigen load and the tumor mutational burden [16,17]; hence, it is essential to investigate possible mechanisms through which the tumor can escape or antagonize the immunotherapeutic efficacy of these mAbs. In this context, a crucial issue is represented by the identification of novel biomarkers predictive of response to ICI treatment. Among several factors under study as potential biomarkers associated with tumor responses to ICIs, the soluble form of PD-L1 (sPD-L1) has gained particular attention due to its identified role as a poor prognostic factor in several cancer types, including multiple myeloma, diffuse large B-cell lymphoma, renal cell carcinoma, and hepatocellular, lung, and gastric cancer [18]. sPD-L1 in blood samples has been suggested to derive from the loss of the transmembrane domain [19], or from the release of extracellular vesicles [20] by both immune and tumor cells. Recently, some evidence has supported the involvement of different pathways in the regulation of PD-L1 expression; however, the most supported ones are the interferon-gamma (IFN-γ) and the Hippo pathways, which regulate PD-L1 mRNA expression through the activation of interferon regulatory factor 1 (IRF1) transcription factor [21], and the interaction of Yes-associated protein (YAP)/PDZ-binding motif (TAZ) with the PD-L1 promoter, respectively [22]. Even if the biological function of sPD-L1 has not been completely defined, an immune suppressive role for sPD-L1 retaining PD-1 binding domain has been described [19,23]. Although the negative association of sPD-L1 with overall survival (OS) has been widely demonstrated in chemotherapy-treated cancer patients [24,25,26,27,28], only a few studies have investigated its role in ICI-treated cancer patients [29], and no data are available for mesothelioma patients. Specifically, high baseline levels of sPD-L1 were found to be negatively associated with clinical benefit in malignant melanoma and non-small cell lung cancer (NSCLC) patients treated with anti-CTLA-4 or anti-PD-1 mAbs, respectively [20,29].

Possible specific mechanisms of regulation carried out by sPD-L1 in relation to different ICIs treatments, as well as their potential clinical implications, need deeper insights. To this end, in this study we exploited the availability of serum samples from an homogeneous cohort of mesothelioma patients, enrolled in the NIBIT-MESO-1 trial (NCT02588131), to investigate the presence and levels of sPD-L1, before and in the course of treatment with the anti-CTLA-4 mAb tremelimumab combined with the anti-PD-L1 mAb durvalumab. Results were broadened by analyzing the effect of anti-CTLA-4, anti-PD-1, and anti-PD-L1 treatment on serum levels of sPD-L1 in a cohort of 81 patients with tumors of different histotypes. Levels of sPD-L1 were correlated with clinical parameters to define their predictive role in ICI therapy.

## 2. Results

### 2.1. Detection of sPD-L1 in NIBIT-MESO-1 Patients 

**Figure 1:**
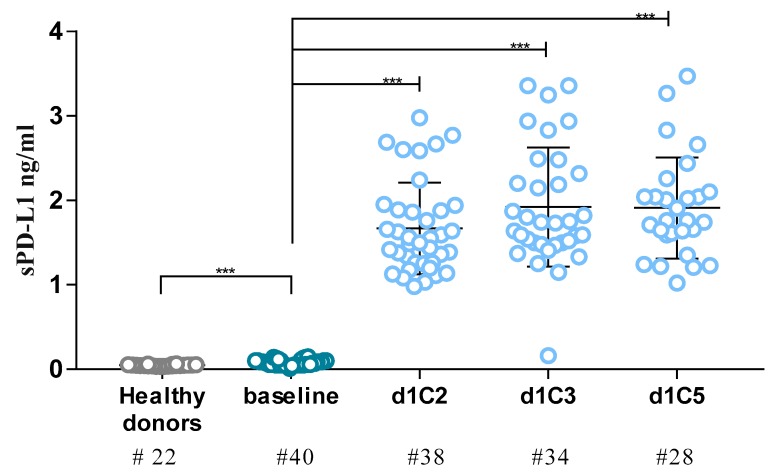
Levels of soluble form of programmed death ligand-1 (sPD-L1) in sera from mesothelioma patients enrolled in the NIBIT-MESO-1 trial and from healthy donors. Levels of sPD-L1 were investigated in sera from 40 mesothelioma patients enrolled in the NIBIT-MESO-1 study by ELISA assay at baseline (dark blue), and during treatment (d1C2, d1C3, d1C5; light blue), and in sera from 22 healthy donors (grey). Each dot represents one patient. *** *p* < 0.001.

Levels of sPD-L1 were determined in sera of 40 mesothelioma patients enrolled in the clinical trial NIBIT MESO-1 at baseline, and of 22 healthy donors. A significant (*p* < 0.001) difference in the mean values of sPD-L1 was observed between mesothelioma patients 0.07 ng/mL (range from 0.01 to 0.15 ng/mL) and healthy donors (0.05 ng/mL; range: 0.03–0.06 ng/mL). To investigate kinetic changes in sPD-L1 levels, sera of NIBIT-MESO-1 patients were analyzed before drug infusion at day 1 of cycle 2 (d1C2), C3, and C5 in the course of treatment, and levels of sPD-L1 were compared to those detected at baseline. Already at d1C2, all patients showed a statistically significant (*p* < 0.001) increase in the sPD-L1 levels, with respect to baseline, that was maintained in the course of treatment with median values of sPD-L1 concentration and fold change vs. baseline (FC) at each investigated time-point ranging from 1.52 ng/mL (d1C2) to 1.76 ng/mL (d1C5), and from 22.71 (d1C2) to 27.28 (d1C3), respectively (Figure 1, Table 1).

### 2.2. Correlation of sPD-L1 Levels with Clinical Parameters in NIBIT-MESO-1 Patients

**Figure 2:**
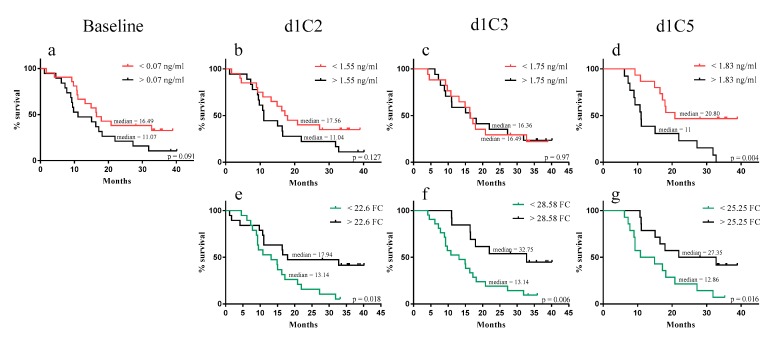
Survival curves of NIBIT-MESO-1 patients generated by Kaplan–Meier analyses. The best cut-off for sPD-L1 concentrations (**a**–**d**) and for FC values (**e**–**g**) post-treatment vs. baseline, defined by receiver operating characteristic (ROC) curve analyses, were used to stratify patients for Kaplan–Meier analyses at baseline (**a**) and at different treatment time-points analyzed (**b**–**g**). Red and black curves represent patients with sPD-L1 concentration below or above the cut-offs identified, respectively (**a**–**d**); green and black curves identified patients with sPD-L1 FC values below or above the cut-offs identified, respectively (**e**–**g**).

The correlation between levels of sPD-L1 and clinical outcome of mesothelioma patients enrolled in the NIBIT-MESO-1 clinical trial was investigated to assess the potential role of sPD-L1 as a predictive biomarker of response to ICI therapy. Receiver operating characteristic (ROC) analyses were utilized to identify the optimal sPD-L1 concentration and FC value cut-offs able to stratify patients according to their OS (Table S1). Results demonstrated that NIBIT-MESO-1 patients with serum concentrations of sPD-L1 below the optimal cut-offs, calculated for each investigated time-point, showed a longer OS at baseline (16.49 vs. 11.07 months), at d1C2 (17.56 vs. 11.04 months), and at d1C5 (20.80 vs. 11.00 months, *p* = 0.004) (Figure 2a,b,d; Table S1). No association between OS and concentration of sPD-L1 resulted at d1C3 (Figure 2c; Table S1).

On the other hand, sPD-L1 FC were significantly associated with OS at any of the time-points analyzed. Specifically, a longer OS of 17.94 vs. 13.14 months (*p* = 0.018) at d1C2, 32.75 vs. 13.14 months (*p* = 0.006) at d1C3, and 27.35 vs. 12.86 months (*p* = 0.016) at d1C5 was observed for patients with sPD-L1 FC values higher than the best cut-offs identified by ROC curves (Figure 2e–g; Table S1).

**Figure 3:**
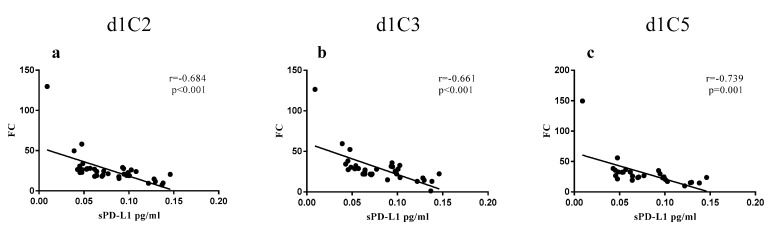
Correlations between sPD-L1 concentrations and FC values in NIBIT-MESO-1 patients. sPD-L1 concentrations detected in sera of NIBIT-MESO-1 patients at baseline were referred to sPD-L1 post-treatment FC values at d1C2 (**a**), d1C3 (**b**), and d1C5 (**c**). Each dot represents one patient.

This opposite trend of Kaplan–Meyer curves is justified by the significant negative correlation observed comparing the concentrations of sPD-L1, at baseline, to the FC values of the soluble protein at each of the investigated time-points (Figure 3).

**Figure 4:**
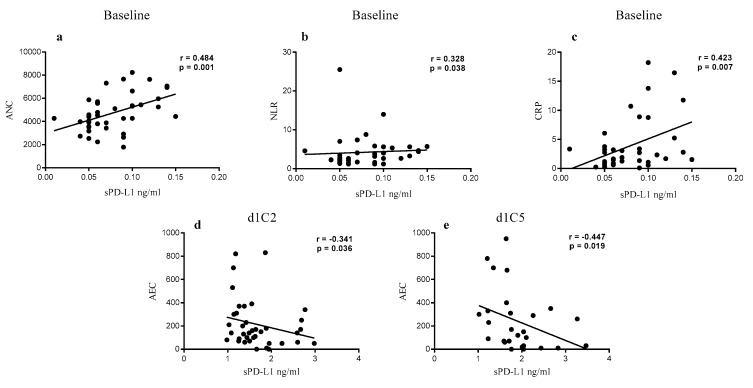
Correlation between hematological parameters and sPD-L1 levels of NIBIT-MESO-1 patients. Levels of sPD-L1 detected in sera from NIBIT-MESO-1 patients were correlated to ANC (**a**), NLR (**b**), and CRP (**c**) at baseline and to AEC at d1C2 (**d**) and d1C5 (**e**). Each dot represents one patient. ANC = absolute neutrophil count; NLR = neutrophil/lymphocyte ratio; CRP = C reactive protein; AEC = absolute eosinophil count.

In addition, to reveal any implication of sPD-L1 in the systemic inflammatory status of cancer patients, blood-derived parameters measured at different time-points were correlated to the levels of sPD-L1 (Table S2). At baseline, among all parameters analyzed, a significant positive correlation was found between sPD-L1 levels and: (i) absolute neutrophil count (ANC) (*r* = 0.48, *p* < 0.01); (ii) neutrophil-to-lymphocyte ratio (NLR) (*r* = 0.33, *p* = 0.04); and (iii) C-reactive protein (CRP) concentrations (*r* = 0.42, *p* < 0.01) (Figure 4a–c; Table S2). A significant positive correlation also existed between the sPD-L1 levels and the eastern cooperative oncology group performance status (ECOG PS), at baseline (*r* = 0.42, *p* < 0.01) (data not shown). In the course of treatment, a significant negative correlation was identified between sPD-L1 and the absolute eosinophil count (AEC) at d1C2 (*r* = −0.34, *p* = 0.04) and d1C5 (*r* = −0.45, *p* = 0.02) (Figure 4d,e; Table S2). 

Finally, no significant correlation was observed between sPD-L1 levels and overall response rate (ORR); immune-related ORR; disease-control rate (DCR); immune-related DCR; or patients age, gender, or clinical stage at all investigated time points.

### 2.3. Levels of sPD-L1 in Patients Treated with Anti-PD-L1 or Anti-CTLA-4 mAbs Monotherapy

Under the assumption that the increase of sPD-L1 levels in NIBIT-MESO-1 patients could be related to treatment, and to discriminate the role of specific ICIs in this phenomenon, levels of the soluble protein were evaluated in serum of additional solid cancer patients (#49), treated with anti-PD-L1 or anti-CTLA-4 in monotherapy. Detection of sPD-L1 was performed at baseline and at specific time-points of treatment.

**Figure 5:**
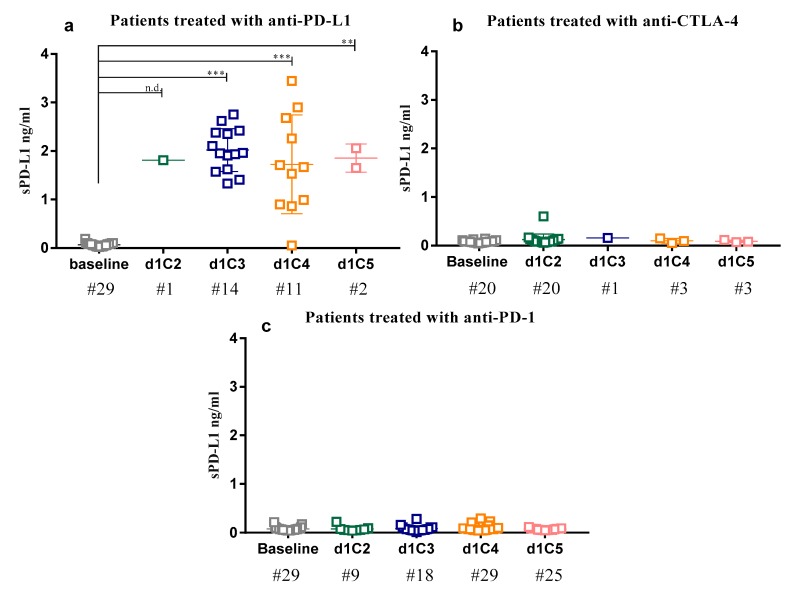
Detection of sPD-L1 in patients treated with different immune check-point inhibitors (ICIs) in monotherapy. Levels of sPD-L1 were determined by ELISA assay in patients treated with anti-PD-L1 (**a**), anti-cytotoxic T-lymphocyte antigen (CTLA)-4 (**b**), or anti-PD-1 (**c**) monoclonal antibodies (mAbs) at baseline (grey), d1C2 (green), d1C3 (blue), d1C4 (orange), and d1C5 (pink). Each dot represents one patient. ** *p* < 0.01; *** *p* < 0.001; n.d., not detectable.

Consistent with the results obtained from NIBIT-MESO-1 study, a statistically significant (*p* < 0.01) increase of sPD-L1 levels was observed in the course of treatment with anti-PD-L1 mAb, in all investigated patients with median values of sPD-L1 FC increasing at each investigated time-point vs. baseline, ranging from 18.25 at d1C4 to 43.9 at d1C3 (Figure 5; Table 2). In detail, the median levels of sPD-L1 at baseline (0.07 ng/mL) increased after the first dose of treatment, reaching its highest values (1.96 ng/mL) at d1C3. Consistently, median values of sPD-L1 FC increased at each investigated time-point vs. baseline, ranging from 18.25 at d1C4 to 43.9 at d1C3.

Patients treated with anti-CTLA-4 mAbs, by contrast, did not show any significant modulation in sPD-L1 serum levels during treatment, with median values of sPD-L1 FC ranging from 0.85 at d1C4 to 1.76 at d1C3 (Figure 5; Table 2).

### 2.4. sPD-L1 Assessment in Sera from Anti-PD-1-Treated Patients

In order to assess whether the increment of sPD-L1 in patients’ sera was specifically related to the administration of anti-PD-L1 mAbs or associated to the blockade of the PD-1/PD-L1 axis, the presence of the soluble protein was investigated in sera from another set of solid cancer patients (#29) treated with anti-PD-1 mAbs. Results showed that median values of sPD-L1 FC, in these patients, did not significantly change between baseline and the following administration cycles, ranging from 1.05 at d1C3 and d1C4 to 1.12 at d1C2 (Table 3).

### 2.5. sPD-L1 Levels in Patients Treated with Anti-PD-L1 

**Figure 6:**
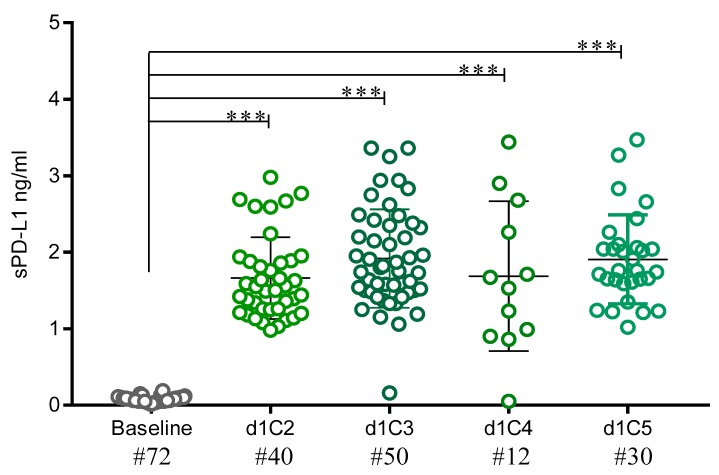
sPD-L1 levels in sera from patients treated with anti-PD-L1. The data plot shows the sPD-L1 levels detected by ELISA assay in sera from 72 cancer patients treated with anti-PD-L1 mAbs, either at baseline (grey) or at d1C2, d1C3, d1C4, and d1C5 (gradations of green). Each dot represents one patient. *** *p* < 0.001.

To strength the statistical reliability of the previously obtained results, analyses of sPD-L1 levels were performed by combining the data obtained from all cancer patients treated with anti-PD-L1, alone or in combination with anti-CTLA-4, reaching a total of 72 patients. Results showed a statistically significant (*p* < 0.001) increment in sPD-L1 concentration after the first cycle of treatment (from median value of 0.06 ng/mL, at baseline, to 1.50 ng/mL) that was maintained through the following cycles of administration reaching the highest median value (1.78 ng/mL) at d1C3. Consistently, median values of sPD-L1 FC increased at each investigated time-point vs. baseline, ranging from 19.77 at d1C4 to 28.58 at d1C3 (Figure 6; Table 4). 

**Figure 7:**
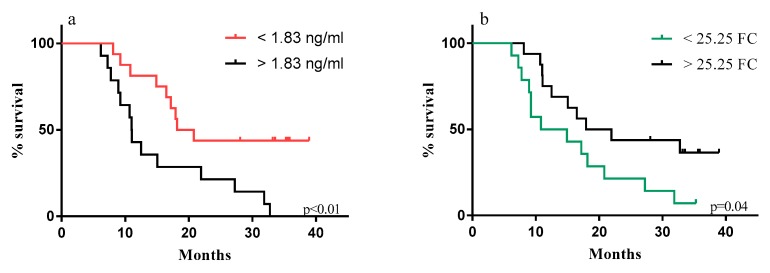
Survival curves of anti-PD-L1-treated patients generated by Kaplan–Meier analyses. The best cut-off values for sPD-L1 concentrations (**a**) and for FC values (**b**), defined by ROC curve analyses, were used to stratify patients for Kaplan–Meier analyses at d1C5. Red and black curves represent patients with sPD-L1 concentrations below or above the cut-off identified (**a**); green and black curves identify patients with FC values below or above the optimal cut-off (**b**).

However, the best cut-off identified at each time-point analyzed, both for FC and concentration values, did not homogeneously stratify patients according to their OS (Table S3). Patients with concentration levels below the identified cut-offs showed a longer survival only at d1C2 and d1C5 (*p* = 0.01) (Figure 7; Table S3). On the other hand, the FC values over the determined cut-offs conferred a longer survival at d1C2, d1C4, and d1C5 (Figure 7; Table S3).

Specifically, the cut-offs of 1.83 ng/mL and of 25.2 FC determined at d1C5 efficiently discriminated patients with a significantly longer OS (*p* < 0.01 and *p* = 0.04, respectively) (Figure 7; Table S3).

Baseline levels of sPD-L1 were correlated with different clinical parameters. The Spearman’s rank correlation was performed to investigate a causal relationship between sPD-L1 and age, gender, stage, eastern cooperative oncology group performance status (ECOG PS), and the overall response rate without observing a statistical significance.

## 3. Discussion

The status of PD-L1 expression on tumor tissue from cancer patients has been the most studied and used as a predictor of efficacy for PD-1/PD-L1 blockade therapy. Although the results collected from different clinical trials [30,31,32,33,34,35] have made the role of PD-L1 expression in tumors rather controversial, it still represents the only predictive biomarker approved into clinical practice for PD-1/PD-L1 blocking therapy [36]. More recently, respect to healthy donors, higher levels of the soluble form of PD-L1 were documented in sera from different cancer patients, correlating with a worse clinical outcome of these patients [27,37,38]. In line with these findings are the results of our study, in which we observed a significantly (*p* < 0.001) higher sPD-L1 concentration in mesothelioma patients compared to healthy donors. Considering the immune suppressive role of PD-L1, these data represent evidence of the predominant immunosuppressive contexture detected in cancer patients compared to healthy donors. Of note, levels of circulating sPD-L1 seem to be heterogeneous in cancer patients; specifically, our results showed that levels of sPD-L1 detected in mesothelioma patients were lower compared to those reported by other studies in cancer patients of different tumor histotypes, such as gastric cancer, B-cell lymphoma, and multiple myeloma [27,38,39]. Further studies are necessary to validate our data because, to the best of our knowledge, this is the first study reporting sPD-L1 levels in mesothelioma patients.

To date, limited information is available in order to describe the mechanism(s) of sPD-L1 release, and it is still unclear whether levels of sPD-L1 may have a predictive role in the course of ICI therapy. To this end, in this study, we investigated this molecule in a cohort of mesothelioma patients treated with the anti-CTLA-4 mAb tremelimumab combined with the anti-PD-L1 mAb durvalumab, within the NIBIT-MESO-1 trial (NCT02588131). Supporting the possible role of this soluble protein as a predictive biomarker of response to ICI therapy, an inverse correlation between concentrations of sPD-L1 and a longer OS was observed at baseline. This observation is consistent with results previously described in other tumor histotypes. Indeed, low levels of sPD-L1 at baseline correlated with a better clinical benefit of NSCLC patients treated with anti-PD-1 mAb [29] and with a longer time before the progression of disease in melanoma patients treated with the anti-CTLA-4 mAb ipilimumab [19]. This inverse correlation between sPD-L1 concentration at baseline and OS of patients might be associated with the active role of sPD-L1 in the suppression of the immune response, as independently described by Zhou and Frigola [19,40]. Intriguingly, the predictive potential of sPD-L1 at baseline as a biomarker of response to ICI therapy seems to be independent of the PD-L1 expression pattern in tumor tissues; in fact, baseline tumor PD-L1 expression has already been reported as being not correlated with ORR or OS of NIBIT-MESO-1 patients [13].

Moreover, considering the evolution of the soluble protein, during ICI therapy, a significant increase of sPD-L1 levels was observed in patients immediately after the first cycle. It is noteworthy to report that sPD-L1 FC vs. baseline, already measured after the first cycle of therapy, were found to be significantly associated to a longer OS of mesothelioma patients, providing fundamental support to the role of changes in circulating sPD-L1 as a non-invasive biomarker for an early prediction of response to immunotherapy. Of note, the direct association observed between patients with a FC over the best cut-off at each time-point and the significant longer survival was strictly dependent upon the baseline level of sPD-L1 of these patients, as confirmed by the significant correlation found between higher FC of sPD-L1 during ICI therapy and the lower baseline concentration. The correlation between constitutive levels of circulating sPD-L1, their FC during anti-PD-L1 therapy, and OS of cancer patients remains to be fully clarified. Along this line, the activation status of immune system induced by ICI therapy could be involved in the shedding of sPD-L1, contributing to the high FC of sPD-L1 released in patients with a longer OS. Although the exact origin of sPD-L1 in cancer patients remains unclear, significant correlations, observed between increase of circulating sPD-L1 levels and fall of PD-L1 expression on both CD4+ (*R^2^* = 0.937, *p* = 0.007) and CD8+ (*R^2^* = 0.91, *p* = 0.012) T-cells in NIBIT-MESO-1 patients (Calabrò et al., manuscript in preparation), suggest that immune cell-surface expression of PD-L1 could be a source of the sPD-L1 detected in our cohort of patients. Moreover, the anti-PD-L1 treatment, inducing an activation of immune response, could increase the release of IFN-γ by effector cells rising the levels of sPD-L1 in patients’ sera [41]. 

A potential association between circulating sPD-L1 and inflammatory response, in cancer patients, is suggested by the positive correlation of sPD-L1 levels with ANC, NLR, and CRP, at baseline, and the negative correlation between the soluble protein and AEC, observed during therapy, in NIBIT-MESO-1 patients. The latter could reflect the distinct roles played by the two variables in cancer patients—the anti-tumorigenic role of eosinophils, emerging in different human tumors such as colorectal, oral, and breast cancer, also associated to a long survival of melanoma patients in response to ICI therapy, and the immune suppressive role of sPD-L1, associated with shorter OS of cancer patients [38,42].

Interestingly, analyzing sera from additional cancer patients treated with anti-CTLA-4, anti-PD-1, or anti-PD-L1 mAbs in monotherapy, we showed that a significant increase of sPD-L1 was detected only in patients treated with anti-PD-L1 but not with anti-CTLA-4 or anti-PD-1 mAbs. This evidence suggests that the increase of sPD-L1 was specifically associated with the blockade of PD-L1 and not generally to the blockade of PD-1/PD-L1 pathway or other immune check-point molecules. Moreover, these results were independent from the treatment schedules, type of anti-PD-L1 mAb, or tumor histotype studied. Our findings are consistent with the analyses of sPD-L1 performed in NSCLC or gastric cancer patients treated with anti-PD-1 mAb [43]. On the other hand, data observed in melanoma patients treated with anti-CTLA-4 or anti-PD-1 therapy showed that the increase of sPD-L1 with a FC > 1.5, after 5 months of treatment, was associated with a favorable clinical outcome [19]. Conversely, although performed in a limited number of patients, our results did not demonstrate a significant increase in sPD-L1 levels in melanoma treated with either anti-PD-1 or CTLA-4 mAbs (data not shown). These different results were possibly due to different biological samples analyzed (plasma vs. sera), to a different sensitivity of the detection system utilized for the measurement of sPD-L1, and/or to a different time of blood sample collection from ICI-treated patients. These hypotheses strengthen the importance of standardization of procedures for a correct sPD-L1 dosage. Moreover, although a slight rise of sPD-L1 was observed in our experimental setting analyzing cancer patients treated with anti-CTLA-4 and anti-PD-1, these levels were not comparable with the stronger increases observed in anti-PD-L1-treated patients. 

The involvement of anti-PD-L1 mAbs in the increase of sPD-L1, regardless of tumor histotypes or treatment schedules, was enforced by the comprehensive analysis performed on all patients treated with anti-PD-L1 mAbs, alone or in combination. It is also noteworthy that in these patients, sPD-L1 concentration was also associated with a longer OS of patients, but it was evident only at selected time-points during therapy, probably due to different factors that could affect these results (e.g., cancer histotypes, timing, and doses of ICI administration). Additional studies aiming to clearly define this topic in a larger cohort of patients should be performed.

## 4. Materials and Methods

### 4.1. Study Participants

A number of 121 patients diagnosed with tumors of different histotypes and treated with anti-PD-L1, anti-PD-1, or anti-CTLA-4 mAbs were investigated (Figure S1; Table S4). They included 40 mesothelioma patients enrolled in the NIBIT-MESO-1 trial (anti-CTLA-4 mAb tremelimumab combined with the anti-PD-L1 mAb durvalumab) and other 32 solid cancer patients treated with the anti-PD-L1 mAbs atezolizumab or durvalumab alone or in combination with the anti-CTLA-4 mAb tremelimumab; 20 solid cancer patients treated with the anti-CTLA-4 mAbs ipilimumab, or tremelimumab; and 29 solid cancer patients treated with the anti-PD-1 mAbs pembrolizumab, or nivolumab. Twenty-two healthy volunteers were used as a control cohort after signing an informed consent form. This research was approved by an institutional ethic committee on 7 May 2018 (ethic code: 12797_2018).

### 4.2. Biological Specimens

Sera samples obtained from each patient were collected at baseline and before drug infusion at day 1 of cycle 2, 3, 4, and 5 in the course of ICI administrations. Blood samples were collected in serum separation tubes (BD Vacutainer, Franklin Lakes, NJ, USA) and centrifuged at 1600× *g* for 15 min at 4 °C (within 30 min after collection), aliquoted, and subsequently stored at −80 °C. Haematologic blood tests (Table S2) were performed by the central laboratory of the University Hospital of Siena (Siena, Italy).

### 4.3. sPD-L1 Analyses

Levels of sPD-L1 in sera from patients were measured by the enzyme-linked immunosorbent assay (ELISA), using the PD-L1/B7-H1 Quantikine ELISA Immunoassay kit (catalougue number. DB7H10, R&D systems, Minneapolis, MN, USA), according to the manufacturer’s protocol. The optical density of each well was measured with the Benchmark PLUS Microplate spectrophotometer (Bio-Rad, Hercules, CA, USA) at 450 nm with wavelength correction set at 570 nm. sPD-L1 concentrations were extrapolated from a specific six-point standard curve. Results were reported as sPD-L1 concentrations or as FC values. The FC values were calculated as the ratio of sPD-L1 concentrations at each time-point compared to the baseline.

### 4.4. Statistical Analysis

Results were analyzed by descriptive statistics to determine median and concentration ranges of sPD-L1. Changes in sPD-L1 levels between baseline and different time-points during treatment were investigated by the Mann–Whitney U test. Correlations between sPD-L1 levels and the patients’ clinical status, as well as biochemical and peripheral blood count parameters (Table S2), were conducted through Spearman’s rank correlation. Each cohort of patients was divided into two groups according to the median OS calculated from the date of enrollment for each patients and data cut-off of 30 March, 2019, and ROC curve analyses were performed to determine the best (maximum specificity and sensitivity) cut-off values for sPD-L1 concentration and FC. Kaplan–Meier analyses were exploited to estimate survival rates, with two-sided 95% CI calculated on the basis of a normal approximation. Survival curves were compared through the log-rank test. A *p*-value < 0.05 was considered statistically significant. Statistical analyses were carried out by GraphPad Prism 7.05 (GraphPad Software Inc., San Diego, CA, USA).

## 5. Conclusions

In conclusion, we demonstrated that both low baseline levels of sPD-L1 and their high FC increase during therapy significantly correlated with a longer OS of mesothelioma patients enrolled in the NIBIT-MESO-1 study. Moreover, we provided the first piece of evidence that increased shedding of sPD-L1 specifically associates with anti-PD-L1 therapy in cancer patients. Comprehensively, these results warrant further investigation to corroborate the use of sPD-L1 as a predictive biomarker of response to anti-PD-L1 therapy in cancer patients.

## Figures and Tables

**Table 1:** sPD-L1 in sera from NIBIT-MESO-1 patients.

sPD-L1 in NIBIT-MESO-1 Patients						
	No.° of Patients	Median ^a^	Concentration Range ^a^	*p*-Value	Median FC ^b^	FC Range
Baseline	40	0.07	0.01–0.15	–	–	–
d1C2	38	1.52	0.98–2.98	<0.001	22.71	7.15–129.66
d1C3	34	1.73	0.16–3.36	<0.001	27.28	1.16–126.39
d1C5	28	1.76	1.02–3.47	<0.001	25.25	9.94–149.35

^a^ values are expressed as ng/mL; ^b^ FC: fold changes were calculated as the ratio of sPD-L1 concentrations at each time-point during treatment compared to the baseline.

**Table 2:** sPD-L1 in patients treated with anti-PD-L1 or anti-CTLA-4 mAbs.

Anti-PD-L1-Treated Patients						
	No.° of Patients	Median	Concentration Range ^a^	*p*-Value	Median FC ^b^	FC Range
Baseline	29	0.07	0.02–0.19	–	–	–
d1C2	1	1.81	1.81	–	43.41	43.41
d1C3	14	1.96	1.33-2.75	<0.001	43.89	17.59–70.54
d1C4	11	1.67	0.05–3.44	<0.001	18.25	0.71–36.91
d1C5	2	1.85	1.65–2.06	0.002	42.36	32.05–52.67
**Anti-CTLA-4-Treated Patients**						
	**No.° of Patients**	**Median**	**Concentration Range ^a^**	***p*-Value**	**Median FC ^b^**	**FC Range**
Baseline	20	0.08	0.04–0.14	–	–	–
d1C2	20	0.09	0.05–0.60	0.06	1.07	0.78–14.31
d1C3	1	0.16	0.16	–	1.76	1.76
d1C4	3	0.09	0.05–0.15	0.58	0.85	0.83–2.19
d1C5	3	0.08	0.07–0.11	0.75	1.51	0.79–0.93

^a^ Values are expressed as ng/mL; ^b^ FC: fold changes were calculated as the ratio of sPD-L1 concentrations at each time-point vs. baseline.

**Table 3:** sPD-L1 in patients treated with anti-PD-1.

Anti-PD-1-Treated Patients						
	No. Patients	Median	Concentration Range ^a^	*p*-Value	Median FC ^b^	FC Range
Baseline	29	0.07	0.04–0.21	–	–	–
d1C2	9	0.06	0.04–0.22	0.45	1.12	0.95–1.45
d1C3	18	0.07	0.01–0.28	0.78	1.05	0.08–1.94
d1C4	29	0.07	0.04–0.29	0.39	1.05	0.76–2.52
d1C5	15	0.07	0.05–0.12	0.85	1.11	0.76–1.46

^a^ Values are expressed as ng/mL; ^b^ FC: fold changes were calculated as the ratio of sPD-L1 concentrations at each time-point compared to the baseline.

**Table 4:** Global analyses of sPD-L1 in all patients treated with anti-PD-L1, alone or in combination with anti-CTLA-4 mAbs.

Anti-PD-L1-Treated Patients						
	No. Patients	Median ^a^	Concentration Range ^a^	*p*-Value	Median FC ^b^	FC Range
Baseline	72	0.06	0.01–0.19	–	–	–
d1C2	40	1.52	0.98–2.98	<0.001	23.39	7.15–129.66
d1C3	50	1.78	0.16–3.36	<0.001	28.58	1.16–126.39
d1C4	12	1.60	0.05–3.44	<0.001	19.77	0.71–49.78
d1C5	30	1.76	1.02–3.47	<0.001	26.21	9.95–149.35

^a^ Values are expressed as ng/mL; ^b^ FC: fold changes were calculated as the ratio of sPD-L1 concentrations at each time-point compared to the baseline.

## Supplementary Materials

The following are available online at https://www.mdpi.com/2072-6694/12/2/361/s1: Figure S1: Flow chart of patient selection process. Table S1: Statistical analyses in NIBIT-MESO-1 patients. Table S2: sPD-L1 association with peripheral blood leukocyte counts and biochemical parameters. Table S3: Statistical analyses for patients treated with anti-PD-L1 ICIs. Table S4: Clinical and demographic parameters of investigated patients and healthy donors.
