# Supplementary material for: Circulating Levels of PD-L1 in Mesothelioma Patients from the NIBIT-MESO-1 Study: Correlation with Survival
Source: Cancers (Basel). 2020 Feb 5;12(2):361. doi: 10.3390/cancers12020361 (PMC7072596; doi:10.3390/cancers12020361)
Supplement: Supplementary file 1 [file cancers-12-00361-s001.pdf]

SUPPLEMENTARY MATERIAL

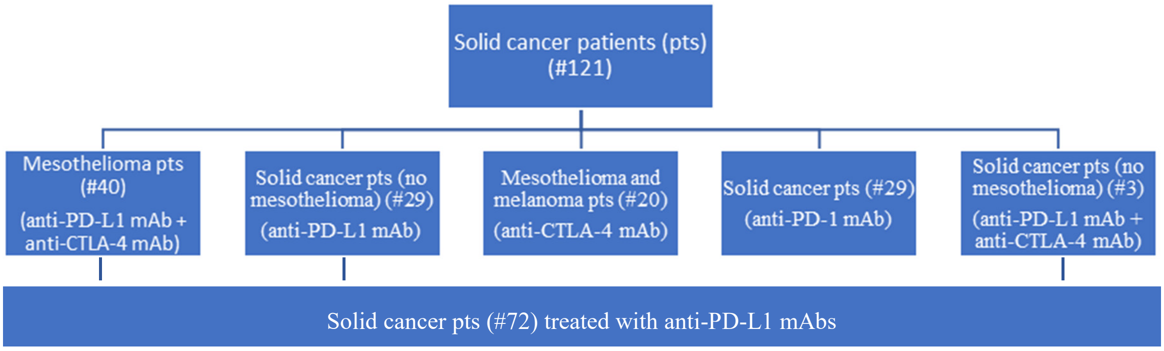

Figure S1. Flow chart of patient selection process.

Figure shows the number of patients investigated in this study stratified according to their treatment.

Table S1. Statistical analyses in NIBIT-MESO-1 patients.

NIBIT-MESO-1 Patients								
		ROC Analyses					Kaplan-Meier Analyses	
	Time-Points	Cut-off	AUC[b]	Sensitivity (%)	Specificity (%)	Likelihood Ratio	OS[c]	*p* Value (Log Rank)
sPD-L1 concentrati	Baseline	0.07[a]	0.61	60.00	55.00	1.33	16.49 vs 11.07	0.09
	d1C2	1.55	0.62	63.16	57.89	1.50	17.56 vs 11.04	0.13
	d1C3	1.75	0.51	58.82	52.94	1.25	16.49 vs 16.36	0.97
	d1C5	1.83	0.61	71.43	64.29	2.00	20.80 vs 11.00	<0.01
FC[d]	d1C2	22.60	0.64	68.42	63.16	1.85	13.14 vs 17.94	0.02
	d1C3	28.58	0.67	58.82	82.35	3.33	13.14 vs 32.75	<0.01
	d1C5	25.25	0.64	64.29	64.29	1.80	12.86 vs 27.35	0.02

[a,] Values are expressed as ng/ml; [b,] AUC: area under the curve; [c,] OS: overall survival of patients with sPD-L1 serum levels below vs. above the optimal cut-off; [d,] FC: fold changes are calculated as the ratio of sPD-L1 concentrations at each treatment time-point *vs* baseline.

Table S2. sPD-L1 association with peripheral blood leukocyte counts and biochemical parameters.

	Variable	Mean ± SD	Conc. Range[a]	Correlation Coefficient (r)	*p* Value
**Baseline**	ALC[b]	1589.00 ± 761.30	230.00–3620.00	−0.08	0.62
	ANC[c]	4732.00 ± 1591.00	1780.00–8240.00	0.48	<0.01
	AMC[d]	738.80 ± 335.30	220.00–2100.00	0.29	0.07
	AEC[e]	163.30 ± 146.90	20.00–650.00	−0.14	0.39
	NLR[f]	4.20 ± 4.30	1.16–25.50	0.33	0.04
	MLR[g]	0.60 ± 0.50	0.15–3.00	0.28	0.08
	LDH[h]	168.70 ± 22.90	119.00–247.00	−0.16	–
	CRP[i]	3.80 ± 4.60	0.10–18.20	0.42	<0.01
**d1C2**	ALC[b]	1524.00 ± 827.30	390.00–4060.00	−0.06	0.72
	ANC[c]	5045.00 ± 1950.00	1300.00–8510.00	−0.09	0.61
	AMC[d]	756.70 ± 321.10	190.00–1660.00	0.02	0.92
	AEC[e]	231.30 ± 232.30	0.00–890.00	−0.34	0.04
	NLR[f]	4.50 ± 3.80	0.87–20.00	−0.03	0.86
	MLR[g]	0.60 ± 0.40	0.13–2.20	0.00	0.99
	LDH[h]	180.10 ± 50.03	114.00–409.00	−0.11	–
	CRP[i]	3.80 ± 4.40	0.07–17.05	−0.16	0.33
**d1C3**	ALC[b]	1662.00 ± 892.60	520.00–4100.00	0.01	0.95
	ANC[c]	5419.00 ± 2445.00	2260.00–12120.00	0.10	0.59
	AMC[d]	778.80 ± 359.90	260.00–2230.00	0.01	0.94
	AEC[e]	245.60 ± 281.50	0.00–1320.00	−0.31	0.08
	NLR[f]	4.30 ± 3.50	0.78–14.20	−0.02	0.92
	MLR[g]	0.60 ± 0.30	0.15–1.30	−0.09	0.61
	LDH[h]	182.30 ± 35.10	131.00–278.00	−0.08	–
	CRP[i]	4.20 ± 5.30	0.06–22.47	−0.08	0.65
**d1C5**	ALC[b]	1602.00 ± 921.30	420.00–4050.00	−0.23	0.25
	ANC[c]	6051.00 ± 2902.00	2650.00–11760.00	0.11	0.57
	AMC[d]	702.50 ± 297.10	330.00–1460.00	−0.17	0.40
	AEC[e]	234.30 ± 259.00	0.00–950.00	−0.45	0.02
	NLR[f]	5.30 ± 4.60	0.96–19.30	0.21	0.30
	MLR[g]	0.50 ± 0.20	0.20–1.30	0.07	0.72
	LDH[h]	181.70 ± 55.80	117.00–357.00	0.27	–
	CRP[i]	3.70 ± 4.00	0.05–17.54	0.19	0.34

[a,] Values are expressed as: cells/μl (b-g), IU/L (h), and mg/L (i); ALC: absolute lymphocyte count; ANC: absolute neutrophil count; AMC: absolute monocyte count; AEC: absolute eosinophil count; NLR: neutrophil-lymphocyte ratio; MLR: monocyte-lymphocyte ratio; LDH: lactate dehydrogenase; CRP: C-reactive protein.

Table 3. Statistical analyses for patients treated with anti-PD-L1 ICIs.

	All anti-PD-L1-Treated Patients							
				ROC Analyses			Kaplan-Meier Analyses	
	**Time-Point**	**Cut-off**	**AUC[b]**	**Sensitivity (%)**	**Specificity (%)**	**Likelihood Ratio**	**OS[c]**	***p* Value (Log Rank)**
**Concentrations**	**Baseline**	0.06[a]	0.54	55.56	55.56	1.25	10.91 vs 13.85	0.79
	**d1C2**	1.55	0.60	60.00	55.00	1.33	17.18 vs 11.00	0.18
	**d1C3**	1.60	0.50	64.00	44.00	1.14	11.98 vs 15.71	0.23
	**d1C4**	1.38	0.58	83.33	66.67	2.50	4.99 vs 14.32	0.45
	**d1C5**	1.83	0.68	73.33	66.67	2.20	19.49 vs 11.04	*<0.01*
**FC[d]**	**d1C2**	24.05	0.59	55.00	60.00	1.37	13.14 vs 19.49	0.09
	**d1C3**	31.30	0.50	60.00	44.00	1.07	14.91 vs 13.99	0.21
	**d1C4**	19.80	0.52	66,67	66.67	2.00	4.06 vs 13.54	0.62
	**d1C5**	25.25	0.52	60.00	53.33	1.29	12.86 vs 19.95	*0.04*

[a,] Values are expressed as ng/ml; [b,] AUC: area under the curve; [c,] OS: overall survival with sPD-L1 serum levels below *vs* above the optimal cut-off; [d,] FC: fold changes are calculated as the ratio of sPD-L1 concentrations at each time-point compared to the baseline; n.d., not detectable because more than 50% of patients were still alive at the time of analysis.

Table S4. Clinical and demographic parameters of investigated patients and healthy donors.

Anti-PD-L1 Treated Patients			
**Parameters**	**NIBIT-MESO-1 Patients**	Patients Treated in Monotherapy	Patients Treated in Combination with Anti-CTLA-4 mAb
Patients #	40	29	3
Gender, m/f (%)	29/11 (72/28)	19/10 (66/34)	3/0(100/0)
Age, median (range)	66 (42-83)	61 (35-78)	69 (35/81)
Clinical stages	III-IV	IIb-IV	III-IV
Tumor histotype	mesothelioma	melanoma, ovarian cancer, breast cancer, leiomyosarcoma, non-small cell lung cancer, neuroendocrine tumor, parotid tumor, colorectal cancer, cervical cancer, testicular cancer, thymoma, bladder cancer, urothelial cancer	head and neck cancer, lung cancer, non-small cell lung cancer

**Anti-PD-1 Treated Patients**	
	Parameters
Patients #	29
Gender, m/f (%)	15/14 (52/48)
Age, median (range)	64 (28-77)
Clinical stages	III-IV
Tumor histotype	neuroendocrine tumor, mesothelioma, ovarian cancer, endometrial cancer, prostatic cancer, bile duct cancer, glioblastoma, renal carcinoma, meningocerebral cancer, melanoma, lung cancer

**Anti-CTLA-4 treated patients**	
	Parameters
Patients #	20
Gender, m/f (%)	11/9 (55/45)
Age, median (range)	66 (37-80)
Clinical stages	III-IV
Tumor histotype	melanoma, mesothelioma

**Healthy donors**	
	Parameters
Patients #	22
Gender, m/f (%)	9/13 (41/59)
Age, median (range)	41 (25-65)
